# SLAM Project - Long-Term Ecological Study of the Impacts of Climate Change in the natural forest of Azores: VII - Long-term arthropod monitoring in Graciosa Island

**DOI:** 10.3897/BDJ.14.e194216

**Published:** 2026-06-26

**Authors:** Martha Vounatsi, Sébastien Lhoumeau, Alexandra Dal Lago, Sophie Wallon, Luís Carlos Fonseca Crespo, Carlos F. S. Picanço, Pedro M. L. Raposo, Paulo A. V. Borges

**Affiliations:** 1 University of Athens, Athens, Greece University of Athens Athens Greece; 2 University of Azores, CE3C—Centre for Ecology, Evolution and Environmental Changes, Azorean Biodiversity Group, CHANGE —Global Change and Sustainability Institute, School of Agricultural and Environmental Sciences, Rua Capitão João d’Ávila, Pico da Urze, 9700-042, Angra do Heroísmo, Azores, Portugal University of Azores, CE3C—Centre for Ecology, Evolution and Environmental Changes, Azorean Biodiversity Group, CHANGE —Global Change and Sustainability Institute, School of Agricultural and Environmental Sciences, Rua Capitão João d’Ávila, Pico da Urze, 9700-042 Angra do Heroísmo, Azores Portugal; 3 University of Bologna, Bologna, Italy University of Bologna Bologna Italy; 4 Serviço de Ambiente e Ação Climática da Graciosa, Rua Eng.º Manuel Rodrigues de Miranda, n.º 9-A, 9880-376, Santa Cruz da Graciosa, Azores, Portugal Serviço de Ambiente e Ação Climática da Graciosa, Rua Eng.º Manuel Rodrigues de Miranda, n.º 9-A, 9880-376 Santa Cruz da Graciosa, Azores Portugal; 5 IUCN SSC Atlantic Islands Invertebrate Specialist Group, Angra do Heroísmo, Azores, Portugal IUCN SSC Atlantic Islands Invertebrate Specialist Group Angra do Heroísmo, Azores Portugal; 6 IUCN SSC Monitoring Specialist Group, Angra do Heroísmo, Azores, Portugal IUCN SSC Monitoring Specialist Group Angra do Heroísmo, Azores Portugal

**Keywords:** Arthropoda, Azores, introduced species, long-term monitoring, native forest, SLAM traps, species records

## Abstract

**Background:**

The data we present are part of the long-term project SLAM – Long Term Ecological Study of the Impacts of Climate Change in the natural forest of Azores, which was established in 2012 to monitor arthropod communities in Azorean forest habitats using standardised long-term ecological sampling. The main aim of this project is to understand how major biodiversity erosion drivers, including habitat degradation, biological invasions and climate-related pressures, affect the distribution, abundance and diversity of Azorean arthropods through time. Long-term monitoring is particularly relevant on oceanic islands, where biodiversity change may be expressed more strongly through species turnover, shifts in community composition and increases in introduced taxa than through immediate declines in total species richness. The SLAM framework also contributes to the early detection and documentation of new species occurrences, thereby improving baseline knowledge for conservation planning, biodiversity assessment and biosecurity. Sampling relies on passive flight-interception SLAM (Sea, Land and Air Malaise) traps, which are operated continuously and serviced at regular intervals to provide comparable seasonal and interannual samples.

**New information:**

We sampled 17,064 specimens from samples from the years 2015-2021 out of which 16,491 were identified to the level of species (96.6%) or even subspecies (326 individuals) and 247 were identified to the order, family or genus level. The identified specimens belong to 17 unique orders, 82 families, 156 genera and 165 species and eight subspecies. Of these species and subspecies, 14 were endemic, 49 were native non-endemic, 86 were introduced and 24 have uncertain colonisation status.

A total of 26 species were recorded for the first time on Graciosa Island, but none represents a new record for the Azores Archipelago. Amongst these newly-recorded species, two are Azorean endemics, five are native non-endemics, 16 are introduced and three have uncertain colonisation status, with most detected at very low abundance.

New island records on Graciosa Island comprise nine spiders (Order Araneae), including two Azorean endemic spiders recorded for the first time on Graciosa, *Neon acoreensis* (jumping spider; Salticidae) and *Savigniorrhipis acoreensis* (dwarf sheet spider; Linyphiidae). The remaining newly-recorded spiders are: *Agyneta decora* (dwarf sheet spider; Linyphiidae), *Enoplognatha mandibularis* (cobweb spider; Theridiidae), *Haplodrassus signifier* (ground spider; Gnaphosidae), *Neriene clathrata* (sheetweb spider; Linyphiidae), *Orchestina furcillata* (goblin spider; Oonopidae), *Theridion melanostictum* (cobweb spider; Theridiidae) and *Zelotes aeneus* (ground spider; Gnaphosidae).

New records also include eight beetles (Coleoptera): *Amischa forcipata* (rove beetle; Staphylinidae), *Brassicogethes aeneus* (pollen beetle; Nitidulidae), *Carpelimus troglodytes troglodytes* (rove beetle; Staphylinidae), *Cathormiocerus curvipes* (weevil; Curculionidae), *Omosita discoidea* (sap beetle; Nitudulidae), *Phyllotreta procera* (flea beetle; Chrysomelidae), *Ptenidium pusillum* (featherwing beetle; Ptiliidae) and *Quedius curtipennis* (rove beetle; Staphylinidae).

Additional new records comprise one millipede (Diplopoda), *Cylindroiulus latestriatus* (julid millipede; Diplopoda, Julida) and three true bugs (Hemiptera): *Buchananiella continua* (minute pirate bug; Anthocoridae), *Empicoris rubromaculatus* (thread-legged bug; Reduviidae) and *Pilophorus confusus* (plant bug; Miridae).

Further additions are one ant (Hymenoptera), *Hypoponera eduardi* (ant; Formicidae), one booklouse (Psocodea), *Lepinotus reticulatus* (booklouse; Trogiidae), two thrips (Thysanoptera), *Anisopilothrips venustulus* (thrips; Thripidae) and *Ceratothrips ericae* (thrips; Thripidae) and one bush-cricket (Orthoptera), *Phaneroptera nana* (katydid/bush-cricket; Tettigoniidae).

## Introduction

Oceanic islands are especially vulnerable to biodiversity loss because their biotas are typically small, highly isolated and rich in endemic species, making them particularly sensitive to habitat destruction, fragmentation and biological invasions (Whittaker et al. 2001, Gillespie et al. 2007, Triantis et al. 2010, Fernández-Palacios et al. 2021). In the Azores Archipelago (Portugal), human colonisation profoundly transformed the original landscape, with native forest being largely replaced by agricultural land, forestry plantations and urban areas (Borges and Hortal 2008, Elias et al. 2016, Borges et al. 2019, Borges et al. 2020). As a result, most remaining native habitats are now small, isolated and embedded in human-dominated matrices, conditions that favour ecological degradation and the spread of exotic species (Elias et al. 2016). In such island systems, biodiversity erosion is often shown not only by species loss, but also by shifts in community composition and increasing representation of introduced taxa (Borges et al. 2020).

These processes are particularly relevant on small islands such as Graciosa, where no true native forest remains and only a small secondary patch dominated by *Erica azorica* persists (Elias et al. 2016). The small size, low elevation and poor quality of this remnant habitat make it especially vulnerable to disturbance and to colonisation by exotic arthropod species from surrounding altered environments (Elias et al. 2016, Lhoumeau et al. 2022). As endemic island species are often habitat specialists with small ecological niches and have limited possibilities for dispersal, continued biodiversity surveys are essential to document current assemblages, detect newly-established exotic species and provide the baseline information needed for conservation actions (Borges et al. 2019, Borges et al. 2020, Oyarzabal et al. 2025a). Graciosa Island is the second smallest island of Azores (61 km^2^), with the lowest maximum elevation 405 m a.s.l. (Forjaz et al. 2004). It is estimated that Graciosa has one of the highest Red List Index of all islands of the Azorean Archipelago (an index measuring trends in species' extinction risk over time, ranging from 1 = species not expected to become extinct soon, to 0 = all species extinct) meaning that it has a lower proportion of threatened species (Oyarzabal et al. 2025b). Nonetheless, it is suspected that this result derives from very early extinction of species endemic to Graciosa island (Oyarzabal et al. 2025b). Moreover, the rise of exotic species in Azorean Archipelago is very alarming (Borges et al. 2020) and could compose a major risk for the remaining endemic biodiversity.

As native forest destruction in the Azores has been associated with a potentially severe extinction debt for endemic arthropods (Triantis et al. 2010), continuous surveys are crucial to document current biodiversity patterns, track ongoing community change, guide conservation actions and reinforce island biosecurity by enabling the early detection and monitoring of exotic species introductions (Borges et al. 2020).

## General description

### Purpose

Within the context of SLAM long-term monitoring project, this publication aimed to gain a deeper understanding of Graciosa's biodiversity in the recent years (as the island has not been surveyed since 2015; Lhoumeau et al. (2022)), to investigate the occurrence and current distribution of introduced and endemic arthropods in these habitats and to detect previously unrecorded species.

### Additional information

SLAM project (Long Term Ecological Study of the Impacts of Climate Change in the natural forest of Azores) started in 2012 for Terceira Island under the project NETBIOME ISLANDBIODIV and one of its goals was to better understand the impact of the drivers of biodiversity in the Azorean Archipelago native forests (Costa and Borges 2021). In August-September 2013, the study continued for seven more islands, namely Flores, Faial, Pico, Graciosa, São Miguel and Santa Maria (Lhoumeau et al. 2022). Since 2020, the long-term monitoring survey is being funded by the project LIFE BEETLES (Lhoumeau et al. 2024), that consists of surveying islands Terceira, Flores and Pico. Even though Graciosa is not part of this funded project, Azorean Government and the Forest Rangers helped to continue to sample monthly or bimonthly.

## Project description

### Title

SLAM - Long Term Ecological Study of the Impacts of Climate Change in the natural forest of Azores: Graciosa Island

### Personnel

The project was conceived and led by Paulo A. V. Borges.

Fieldwork was done by Carlos Picanço with the collaboration of Pedro Raposo (Natural Park of Graciosa).

Parataxonomists that worked on the samples related to this article were: Martha Vounatsi, Alexandra Dal Lago, Sophie Wallon, Alejandro Torres Expósito, Guillaume Péron, José Luis Laguna Fernández, Luna Serrano Navarro, Noelia Reverón García, Yeray Gómez López.

Taxonomic work was held by Paulo A. V. Borges and Luís C. Crespo.

Voucher specimen management was undertaken by Martha Vounatsi, Alexandra Dal Lago, Sophie Wallon, Luís C. Crespo and Paulo A. V. Borges.

Darwin Core database management was currated by Sébastien Lhoumeau and Paulo A. V. Borges.

### Study area description

**Figure 1.**
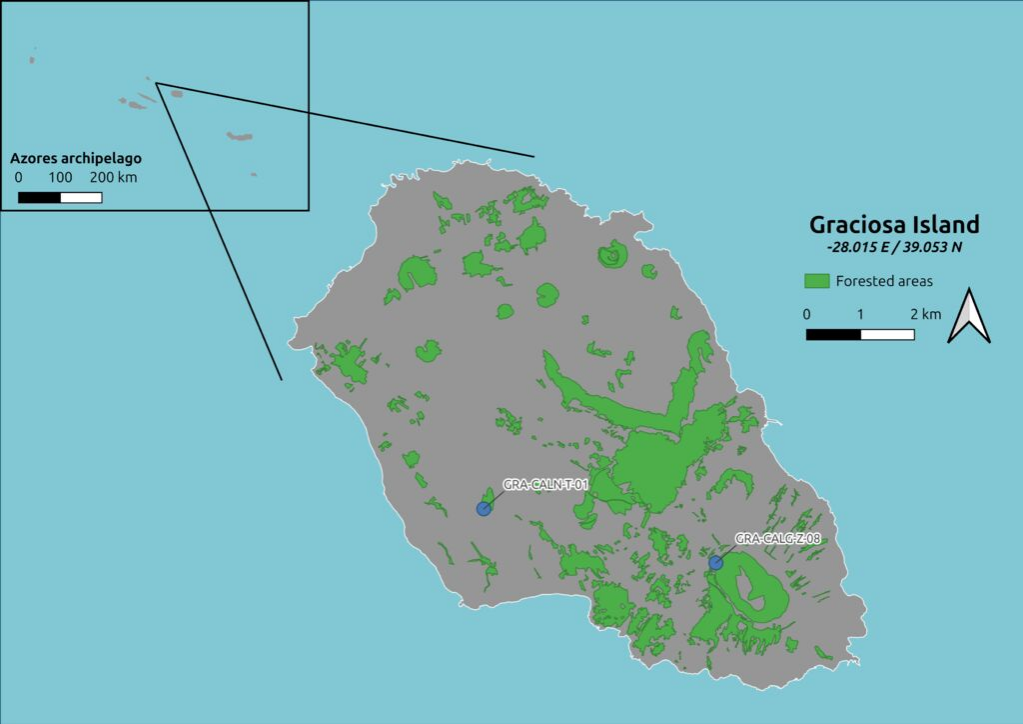
Graciosa Island: Forested areas and sampling areas where traps were located are highlighted. See complete site information in Table 1.

In this study, two sites from Graciosa Island (Azores, Portugal) were sampled. Graciosa is one of the warmest and driest islands of the Azorean Archipelago and it qualifies as Csa in the Köppen Climate Classification with temperate climate with hot and dry summers (Elias et al. 2016). One of the sites (Caldeirinha de Pêro Botelho) consists of *Erica azorica* woodland and is located at 348 m a.s.l. More specifically, it is classified as *Erica-Morella* Coastal woodland, based on Elias et al. (2016). The second one (Caldeira da Graciosa-Furna da Maria Encantada) is an exotic and mixed forest and is located at 227 m a.s.l. (Table 1, Fig. 1).

**Caldeirinha de Pêro Botelho**

The Caldeirinha de Pêro Botelho represents the crater of the only identified spatter cone (lava-splash cone) on Graciosa Island (Açores Geoparque 2026). The crater exhibits a near-circular morphology and provides access to the island’s sole documented volcanic cave (Açores Geoparque 2026). This cave has a vertical extent of approximately 37 m and opens at its base into a subhorizontal gallery orientated roughly NE–SW, with dimensions of 24.6 m in length and 7.4 m in width. The floor of the gallery is largely occupied by angular blocks derived from gravitational collapse of the cavity walls and ceiling.

The volcanic cavity was first systematically explored and documented in 1964 by the Sociedade de Exploração Espeleológica – Os Montanheiros. The surrounding crater rim allows observation of the internal depression as well as the broader geomorphological context, including the Northwest Platform of Graciosa Island. This platform is predominantly basaltic and is characterised by a high density of monogenetic volcanic landforms, comprising approximately 55 cinder cones.

Due to its geological uniqueness and scientific relevance, the Caldeirinha de Pêro Botelho is classified as a priority geosite within the Azores Geopark, with recognised significance at the regional scale for scientific research, geoscience education and geotourism (Açores Geoparque 2026)

**Caldeira da Graciosa-Furna da Maria Encantada**

Furna da Maria Encantada is a natural heritage site within the Graciosa Nature Park and corresponds to a lava tube developed on the slope of the Caldeira da Graciosa. According to Pereira et al. (2015), the cavity is 56.5 m long, with a maximum width of 4.9 m and a maximum height of 5.8 m. In addition to its geological interest, this site provides a privileged view over the interior of the caldera and is included in the walking trail Volta à Caldeira – Furna do Enxofre (Secretaria Regional do Ambiente e Ação Climática, Governo dos Açores 2026).

### Design description

Both sites were sampled monthly or every two months by Forest Rangers, yielding a large dataset that allows a reliable characterisation of the richness and composition of the arthropod communities present in the study areas. Moreover, because these sites include secondary native woodlands and mixed native–exotic forest fragments, they provide an important opportunity to investigate whether small remnant patches of native vegetation can function as refuges for endemic arthropod biodiversity on Graciosa, where this habitat is now highly reduced. Native forest fragments are especially relevant in the Azores because they support the most distinctive indigenous arthropod assemblages and may buffer the impacts of habitat degradation and exotic species spread.

### Funding

FCT-NETBIOME –ISLANDBIODIV grant 0003/2011 (between 2012 and 2015).

EU ERASMUS+ Training Grants to Alejandro Torres Expósito, Alexandra Dal Lago, Guillaume Péron, José Luis Laguna Fernández, Luna Serrano Navarro, Noelia Reverón García, Yeray Gómez López.

Open access was funded by FCT-UID/00329/2025 - Centre for Ecology, Evolution and Environmental Changes (CE3C) DOI https://doi.org/10.54499/UID/00329/2025

Funding in 2026 was obtained from the project “Upgrading the Azorean Biodiversity Portal Infrastructure (AZORES BIOPORTAL- PORBIOTA) to Boost Biodiversity Research, Management and Education -PORBIOTA” (DRCID, ACORES2030-FEDER-03420600).

## Sampling methods

### Study extent

In total, we conducted 117 sampling sessions. Caldeirinha de Pêro Botelho (*Erica azorica* remnants) was sampled 61 times and Furna da Maria Encantada (mixed exotic forest) was sampled 56 times. It is very important that both native and exotic habitats are sampled for further investigation. As Graciosa is quite small in area, two sites are providing representative information about the island's biodiversity.

### Sampling description

**Figure 2.**
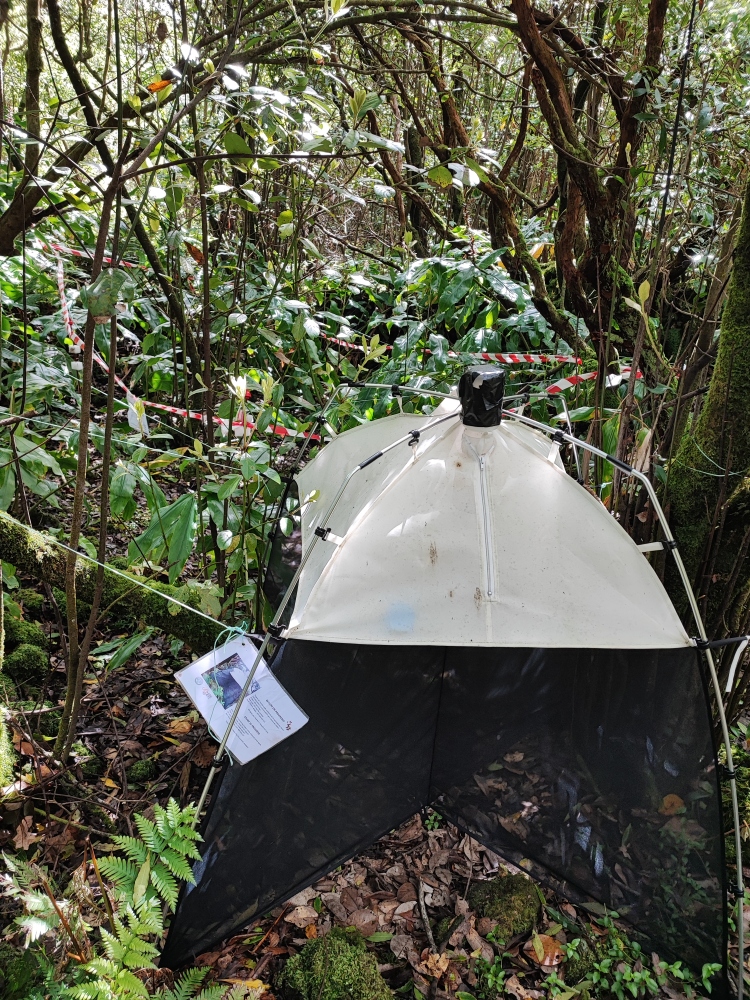
SLAM traps (Sea, Land and Air Malaise trap). Credit: Paulo A. V. Borges.

Arthropods were sampled in native and exotic vegetation plots using passive flight-interception SLAM traps (Sea, Land and Air Malaise trap; 110 cm × 110 cm × 110 cm; MegaView Science Co. Ltd., Taichung City, Taiwan) (Fig. 2), with one trap deployed per plot. In this system, intercepted arthropods crawl upwards along the mesh and fall into a central collecting recipient filled with propylene glycol (pure 1,2-propanediol), which ensures specimen preservation between sampling periods and maintains material suitable for future DNA-based analyses. Although SLAM traps were initially designed for flying arthropods, they also capture non-flying taxa that use the trap structure as an extension of the vegetation. This feature broadens the taxonomic coverage of the method and makes it suitable for monitoring diverse arthropod assemblages.

### Quality control

All specimens were carefully sorted, identified and curated under the close supervision of Paulo A. V. Borges. Taxonomic identification followed standard procedures and was based on external morphological traits and, when required, genitalic characters for accurate species delimitation. Species nomenclature and colonisation status were assigned according to the most recent checklist of the Azorean arthropods (Borges et al. 2022).

### Step description

After sorting and identifying to the most precise taxon as possible, we made a reference collection for all specimens, whether they were identified to the level of species or not. Specimens were assigned morphospecies codes and permanently deposited in the Dalberto Teixeira Pombo Insect Collection (DTP) at the University of the Azores, Terceira, Portugal.

## Geographic coverage

### Description

This survey is focused on the island of Graciosa (Azores, Portugal).

### Coordinates

39.03 and 39.039 Latitude; -28.03 and -27.98 Longitude.

## Taxonomic coverage

### Description

In this study we focus on:

Arachnida: Araneae, Opiliones, Pseudoscorpiones.

Chilopoda: Lithobiomorpha, Scutigeromorpha.

Diplopoda: Julida, Polydesmida.

Insecta: Archaeognatha, Coleoptera, Dermaptera, Hemiptera, Hymenoptera (Formicidae), Lepidoptera, Neuroptera, Orthoptera, Psocodea, Strepsiptera, Thysanoptera.

## Traits coverage

To access traits of some of the species, consult Oyarzabal et al. (2026).

## Temporal coverage

**Data range:** 2015-5-26 – 2022-1-26.

### Notes

Samples were collected monthly or bimonthly with the exception of the period March 2020-June 2020, when there was no collection of samples. A previous study by Lhoumeau et al. (2022) included the first six months of sampling in 2015 and some samples from 2016.

## Collection data

### Collection name

Dalberto Teixeira Pombo insect collection at the University of Azores.

### Collection identifier

DTP 

### Parent collection identifier

DTP

### Specimen preservation method

All specimens were preserved in 96% ethanol. 

### Curatorial unit

Curatorial unit: Dalberto Teixeira Pombo insect collection at the University of the Azores (Curator: Paulo A. V. Borges).

## Usage licence

### Usage licence

Creative Commons Public Domain Waiver (CC-Zero)

## Data resources

### Data package title

Long-term monitoring of Azorean forest arthropods: Graciosa Island.

### Resource link

https://doi.org/10.15468/vhpj9z

### Alternative identifiers

https://www.gbif.org/dataset/8b00b391-704c-43a4-863c-3daec36b00f7

### Number of data sets

2

### Data set 1.

#### Data set name

Event table

#### Data format

Darwin Core

#### Character set

UTF-8

#### Download URL

https://ipt.gbif.pt/ipt/resource?r=slam_graciosa

#### Data format version

1.2

#### Description

The dataset was published in the Global Biodiversity Information Facility platform, GBIF (Borges et al. 2026). The following data table includes all the records for which a taxonomic identification of the species was possible. The dataset submitted to GBIF is structured as a sample event dataset that has been published as a Darwin Core Archive (DwCA), which is a standardised format for sharing biodiversity data as a set of one or more data tables. The core data file contains 124 records (eventID).

**Data set 1.** 

Column label	Column description
id	Unique identification code for sampling event data.
eventID	Identifier of the events, unique for the dataset.
samplingProtocol	The sampling protocol used to capture the species.
sampleSizeValue	The numeric amount of time spent in each sampling (in days).
sampleSizeUnit	The unit of the sample size value.
eventDate	Date or date range the record was collected.
eventRemarks	The verbatim original representation of the date and time information for an Event. In this case, we use the season and year.
habitat	The habitat from which the sample was obtained.
locationID	Identifier of the location.
islandGroup	Name of Archipelago, always Azores in the dataset.
island	Name of the island, always Graciosa in the dataset.
country	Country of the sampling site, always Portugal in the dataset.
countryCode	ISO code of the country of the sampling site, always PT in the dataset.
continent	The name of the continent in which the dcterms:Location occurs, always Europe in this dataset.
municipality	Municipality of the sampling site.
locality	Name of the locality.
minimumElevationInMetres	The lower limit of the range of elevation (altitude, above sea level), in metres.
locationRemarks	Details on the locality site.
decimalLatitude	Approximate decimal latitude of the trap.
decimalLongitude	Approximate decimal longitude of the trap.
geodeticDatum	The ellipsoid, geodetic datum or spatial reference system (SRS), upon which the geographic coordinates given in decimalLatitude and decimalLongitude are based, always WGS84 in the dataset.
coordinateUncertaintyInMetres	Uncertainty of the coordinates of the centre of the sampling plot.
coordinatePrecision	Precision of the coordinates.
georeferenceSources	A list (concatenated and separated) of maps, gazetteers or other resources used to georeference the Location, described specifically enough to allow anyone in the future to use the same resources.
year	The four-digit year in which the dwc:Event occurred, according to the Common Era Calendar.
datasetName	The name identifying the dataset from which the record was derived.

### Data set 2.

#### Data set name

Occurrence table

#### Data format

Darwin Core

#### Character set

UTF-8

#### Download URL

https://ipt.gbif.pt/ipt/resource?r=slam_graciosa

#### Data format version

1.2

#### Description

The dataset was published in the Global Biodiversity Information Facility platform, GBIF (Borges et al. 2026). The following data table includes all the records for which a taxonomic identification of the species was possible. The dataset submitted to GBIF is structured as an occurrence table that has been published as a Darwin Core Archive (DwCA), which is a standardised format for sharing biodiversity data as a set of one or more data tables. The core data file contains 2043 records (occurrenceID).

**Data set 2.** 

Column label	Column description
id	Unique identification code for species abundance data. Equivalent here to eventID.
type	The nature or genre of the resource, as defined by the Dublin Core standard. In our case "PhysicalObject".
licence	Reference to the licence under which the record is published.
institutionID	The identity of the institution publishing the data.
collectionID	The identity of the collection publishing the data.
institutionCode	The code of the institution publishing the data.
collectionCode	The code of the collection where the specimens are conserved.
datasetName	Name of the dataset.
basisOfRecord	The nature of the data record.
recordedBy	A list (concatenated and separated) of names of people, groups or organisations who performed the sampling in the field.
occurrenceID	Identifier of the record, coded as a global unique identifier.
organismQuantity	A number or enumeration value for the quantity of organisms.
organismQuantityType	The type of quantification system used for the quantity of organisms.
sex	The sex and quantity of the individuals captured.
lifeStage	The life stage of the organisms captured.
establishmentMeans	The process of establishment of the species in the location, using a controlled vocabulary: 'native', 'introduced', 'endemic', 'uncertain'.
eventID	Identifier of the events, unique for the dataset.
identifiedBy	A list (concatenated and separated) of names of people, groups or organisations who assigned the taxon to the subject.
dateIdentified	The date on which the subject was determined as representing the taxon.
scientificName	Complete scientific name including author and year.
kingdom	Kingdom name.
phylum	Phylum name.
class	Class name.
order	Order name.
family	Family name.
genus	Genus name.
specificEpithet	Specific epithet.
infraspecificEpithet	Infraspecific epithet.
taxonRank	Lowest taxonomic rank of the record.
scientificNameAuthorship	Name of the author of the lowest taxon rank included in the record.
identificationRemarks	Information about morphospecies identification (code in Dalberto Teixeira Pombo Collection).
occurrenceStatus	A statement about the presence or absence of a dwc:Taxon at a dcterms:Location.

## Additional information

We sampled 17,064 specimens that were successfully identified. Of these, 16,491 were identified to species level (96.6%) or, in some cases, to subspecies level (326), while 247 were identified only to order, family or genus level. We were able to identify such a high percentage of specimens because of the extensive knowledge of Azorean biodiversity gained from more than 25 years of surveying the islands. We were also able to identify a lot of juveniles, even to species level. The identified specimens belong to 17 orders, 82 families, 156 genera and 165 species and eight subspecies. Of these species and subspecies, 14 were endemic, 49 were native non-endemic, 86 were introduced and 24 had an uncertain colonisation status. Although none of the identified specimens constitutes a new record for Azorean Archipelago, we suspect that, amongst them, there is a single-island endemic species that has not yet been described. More specifically, these specimens belong to the genus *Cixius* and the description of this new species would be of considerable scientific interest.

In a previous study in the same series of reports for the long-term monitoring of Azorean arthropod biodiversity, in a short sampling period (2014-2016), 24 species new to the Azorean Archipelago were recorded, mostly introduced and native non-endemic (Lhoumeau et al. 2022). As mentioned above, Graciosa has almost no native vegetation remaining, except for a single patch of *Erica azorica* woodland (Elias et al. 2016) which was sampled in this study. Additionally, the island has a drier and warmer climate than most of the Azorean islands. This represents a warning sign that Graciosa could act as a refuge for introduced species adapted to warmer and drier conditions. During this study, 26 species were recorded for the first time on the island of Graciosa (Table 2), most of which were introduced species (61.5%). Of these 26 species, two were endemic, five were native non-endemic, 16 were introduced and three had an uncertain colonisation status. Most species were found in very low abundance (15 or fewer individuals), except *Hypoponera eduardi*, a native non-endemic species (Forel 1894) which was represented by 42 individuals.

More than 70% of the species, newly recorded during the 2014-2016 survey of Graciosa (Lhoumeau et al. 2022), were recorded again in this study (17 out of 24). One of the most abundant introduced species that was found in the previous study and throughout the years of the present study (2015-2021) was the flatid planthopper *Siphanta acuta* (Walker, 1851). This species is a generalist phytophagous native to Australia, which has invaded multiple regions worldwide, including Vietnam, United States of America: Hawaii and California, South Africa, New Zealand, Chile and the Azorean Archipelago, Portugal (Fletcher 1985, Larivière et al. 2010, Borges et al. 2013). Furthermore, this species can affect crops such as citrus, coffee, banana, guava, mango and eucalyptus (Myers 1922, Zimmerman 1948, Fletcher 1985, Larivière et al. 2010). When these insects are feeding, they secrete a thick honeydew on-to the plant, which subsequently serves as a growth medium for fungi and moulds (Borges et al. 2013). The species was first recorded in the Azores in 2010, in São Miguel Island, at Fenais da Luz and was later observed at Capelas and Ponta Delgada (Borges et al. 2013).

The chrysomelid *Phyllotreta procera* (Redtenbacher, 1849) is present in Central and Eastern Europe, Anatolia, eastern Africa and the Caucasus and throughout Macaronesia (Cabo Verde, Canary Islands and Madeira) (Erber 1986). In the Azores, the species was not recorded until 2019 in Pico (see MF1709 in Borges and Lhoumeau (2024)) using the SLAM protocol as described above and it was not observed on any another Azorean island until this study. The species seems to be spreading to new places in the last 20 years (Molander and Hellqvist 2011). In the present study, specimens of *Phyllotreta procera* were collected in 2015, 2016 and 2020. The initial introduction to the Azorean Archipelago may have occurred via Graciosa; however, due to extensive unsurveyed areas on the islands such as cities and urban parks, this cannot be concluded with certainty.

## Figures and Tables

**Table 1.** Details of the two sampled sites in Graciosa Island where the traps were located with Location ID, locality, decimal coordinates, elevation in metres a.s.l. and habitat according to Elias et al. (2016).

Location ID	Locality	Latitude	Longitude	Elevation	Habitat
GRA-CALN-T-01	Caldeirinha de Pêro Botelho	39.03841	-28.03039	348	*Erica* forest (1)
GRA-CALG-Z-08	Caldeira da Graciosa-Furna da Maria Encantada	39.03003	-27.98049	227	Mixed forest

**Table 2.** List of species newly-recorded for Graciosa Island, with taxonomic information, colonisation status, total number of individuals found and number of individuals found per site.

**Class**	**Order**	**Species Scientific Name**	**Number of Inviduals**	**T01**	**Z08**	**Colonisation Status**
Arachnida	Araneae	*Savigniorrhipis acoreensis* Wunderlich, 1992	1		1	endemic
Arachnida	Araneae	*Neon acoreensis* Wunderlich, 2008	3		3	endemic
Insecta	Coleoptera	*Amischa forcipata* Mulsant & Rey, 1873	1	1		uncertain
Insecta	Coleoptera	*Quedius curtipennis* Bernhauer, 1908	1		1	uncertain
Insecta	Coleoptera	*Carpelimus troglodytes troglodytes* (Erichson, 1840)	1	1		uncertain
Arachnida	Araneae	*Haplodrassus signifer* (C. L. Koch, 1839)	3	3		introduced
Arachnida	Araneae	*Zelotes aeneus* (Simon, 1878)	1	1		introduced
Arachnida	Araneae	*Agyneta decora* (O. Pickard-Cambridge, 1871)	3	3		introduced
Arachnida	Araneae	*Neriene clathrata* (Sundevall, 1830)	3	3		introduced
Arachnida	Araneae	*Orchestina furcillata* Wunderlich, 2008	1		1	introduced
Arachnida	Araneae	*Theridion melanostictum* O. Pickard-Cambridge, 1876	1	1		introduced
Arachnida	Araneae	*Enoplognatha mandibularis* (Lucas, 1846)	1	1		introduced
Diplopoda	Julida	*Cylindroiulus latestriatus* (Curtis, 1845)	2	2		introduced
Insecta	Coleoptera	*Phyllotreta procera* (Redtenbacher, 1849)	5	2	3	introduced
Insecta	Coleoptera	*Omosita discoidea* (Fabricius, 1775)	1	1		introduced
Insecta	Coleoptera	*Brassicogethes aeneus* (Fabricius, 1775)	6	3	3	introduced
Insecta	Coleoptera	*Ptenidium pusillum* (Gyllenhal, 1808)	2	1	1	introduced
Insecta	Hemiptera	*Buchananiella continua* (White, 1880)	2		2	introduced
Insecta	Hemiptera	*Empicoris rubromaculatus* (Blackburn, 1889)	12		12	introduced
Insecta	Psocodea	*Lepinotus reticulatus* Enderlein, 1905	2		2	introduced
Insecta	Thysanoptera	*Anisopilothrips venustulus* (Priesner, 1923)	5		5	introduced
Insecta	Coleoptera	*Cathormiocerus curvipes* (Wollaston, 1854)	3		3	native non-endemic
Insecta	Hemiptera	*Pilophorus confusus* (Kirschbaum, 1856)	4	3	1	native non-endemic
Insecta	Hymenoptera	*Hypoponera eduardi* (Forel, 1894)	42	29	13	native non-endemic
Insecta	Orthoptera	*Phaneroptera nana* Fieber, 1853	2		2	native non-endemic
Insecta	Thysanoptera	*Ceratothrips ericae* (Haliday, 1836)	15	2	13	native non-endemic
